# The Preparation of Graphene Oxide-Silver Nanocomposites: The Effect of Silver Loads on Gram-Positive and Gram-Negative Antibacterial Activities

**DOI:** 10.3390/nano8030163

**Published:** 2018-03-14

**Authors:** Truong Thi Tuong Vi, Selvaraj Rajesh Kumar, Bishakh Rout, Chi-Hsien Liu, Chak-Bor Wong, Chia-Wei Chang, Chien-Hao Chen, Dave W. Chen, Shingjiang Jessie Lue

**Affiliations:** 1 Department of Chemical and Materials Engineering and Green Technology Research Center, Chang Gung University, Taoyuan City 333, Taiwan; truongthituongvi005@gmail.com (T.T.T.V.); rajeshkumarnst@gmail.com (S.R.K.); 2 Department of Biochemical and Biomedical Engineering, Chang Gung University, Taoyuan City 333, Taiwan; bishakh4fun@gmail.com (B.R.); chl@mail.cgu.edu.tw (C.-H.L.); 3 Department of Orthopedic Surgery, Chang Gung Memorial Hospital, Keelung City 204, Taiwan; iborwong@yahoo.com (C.-B.W.); flyinwei@gmail.com (C.-W.C.); chchen1982@gmail.com (C.-H.C.); 4 Department of Safety, Health and Environment Engineering, Ming-Chi University of Technology, New Taipei City 243, Taiwan; 5 Department of Radiation Oncology, Chang Gung Memorial Hospital, Taoyuan City 333, Taiwan

**Keywords:** graphene oxide, silver nanoparticles, thiol groups, antibacterial activity, inhibition efficiencies

## Abstract

In this work, silver nanoparticles (Ag NPs) were decorated on thiol (–SH) grafted graphene oxide (GO) layers to investigate the antibacterial activities in Gram-positive bacteria (*Staphylococcus aureus*) and Gram-negative bacteria (*Pseudomonas aeruginosa*). The quasi-spherical, nano-sized Ag NPs were attached to the GO surface layers, as confirmed by using field emission scanning electron microscopy (FESEM) and transmission electron microscopy (TEM), respectively. The average size of GO-Ag nanocomposites was significantly reduced (327 nm) from those of pristine GO (962 nm) while the average size of loaded Ag NPs was significantly smaller than the Ag NPs without GO. Various concentrations of AgNO_3_ solutions (0.1, 0.2, and 0.25 M) were loaded into GO nanosheets and resulted in the Ag contents of 31, 43, and 65%, respectively, with 1–2 nm sizes of Ag NPs anchored on the GO layers. These GO-Ag samples have negative surface charges but the GO-Ag 0.2 M sample (43% Ag) demonstrated the highest antibacterial efficiency. At 10 ppm load of GO-Ag suspension, only a GO-Ag 0.2 M sample yielded slight bacterial inhibition (5.79–7.82%). As the GO-Ag content was doubled to 20 ppm, the GO-Ag 0.2 M composite exhibited ~49% inhibition. When the GO-Ag 0.2 M composite level was raised to 100 ppm, almost 100% inhibition efficiencies were found on both *Staphylococcus aureus* (S.A.) *and Pseudomonas aeruginosa* (P.A.), which were significantly higher than using pristine GO (27% and 33% for S.A. and P.A.). The combined effect of GO and Ag nanoparticles demonstrate efficient antibacterial activities.

## 1. Introduction

In recent years, antibiotic material development has become disputed due to antibiotic resistance. Antibiotic resistance has spread worldwide and threatens our daily life [1]. Even though the exact mechanism of the antibacterial function is still being exploited, conventional antibiotics have many defects due to inadequate digestion, urinary limitation, and are rapidly losing effectiveness [2]. Antibiotic resistance has been reported to cause genomic structure mutations resulting in bacteria phenotypes changes to reduce antibiotic efficiency and to develop antibiotic resistance [3,4]. 

Recently, many researchers found the benefits of graphene oxide (GO) and versatility in drug delivery and biological resources. GO comprises of a typical two-dimensional material made of carbon atoms which are packed densely in a honeycomb crystal lattice [5] and has been used as a promising material for preparing new composites during the past decades [6]. Moreover, it is reported that GO and its composites possess anti-microbial, anti-bacterial, and anti-fungal agents [7,8]. Several studies have shown the effective antibiotics properties using both physical and chemical mechanisms. Zou et al. claimed that the layer structure of GO can wrap the bacteria cell membrane and cause oxidative stress at the basal plane, thus damaging the cellular membrane [9]. When bacteria membranes are exposed to graphite or GO, the oxidation of glutathione, an important cellular antioxidant, occurs [10]. 

Silver nanoparticles (Ag NPs) are also considered an effective material with antibacterial properties. The bacteria are less prone to develop resistance against Ag NPs than those of conventional antibiotics [11]. Therefore, the combination of Ag NPs and GO is suggested to produce better antibiotic properties than their individual components. The binding between GO and Ag holds good hydrophilicity, high chemical stability, and high oxidization capacity which cause membrane and oxidative stress [12]. The proposed antimicrobial mechanism is that the GO wraps around bacteria while the Ag kills the bacteria with its toxicity [13]. 

Several previous researchers have synthesized GO nanosheets loaded with Ag NPs using the pulsed [14], microwave [15], and sonication methods [16]. Similar to Ag NPs synthesis, GO-Ag NPs preparation also needs a stabilizer and reducing agents. Previous studies have reported some defects of GO-Ag NPs such as aggregation or the formation of inhomogeneous NPs and the large Ag NPs size. For example, Das et al. prepared the GO-Ag NPs using sodium citrate and sodium borohydride (NaBH_4_) as capping and reducing agents [17]. Haider et al. prepared reduced graphene oxide (rGO) doped with Ag NPs using a sequence of AgNO_3_ in aqueous NaBH_4_ as surfactant [18]. Bao et al. reported GO-Ag NPs composites using AgNO_3_ as a salt precursor, hydroquinone as the reducing agent, and citrate as the stabilizer [19]. Yet, the size of Ag NPs was still large (ranging from 20 to 80 nm) and heterogeneously scattered on the GO layers. Furthermore, the major disadvantage of the previously reported methods gives evidence to the difficulty in controlling the size and distribution, limiting the systematic study on the antibacterial effect [20].

A novel method for GO-Ag NPs synthesis is to use NaSH as an effective cross-linker via GO–SH formation [21]. The benefit of binding thiol groups (–SH) [22] is because it is considered a reactive cross-linker and improves the biological compatibility characteristics of the materials [23]. Moreover, the thiol-functionalized GO could improve the particles’ stable suspension in solution to prevent size agglomeration. Besides, GO–SH is an intermediate to bridge the oriented Ag NPs onto the GO’s functional groups, enabling precise particle size control. In this study, our aims are to produce a few nanometer-sized Ag NPs on GO without using extra reducing agents and stabilizers and also to investigate the optimal Ag NPs and GO ratio for high antibacterial activity.

In this study, we fabricated the thiol grafted GO-Ag nanocomposites to investigate their antibacterial activities on *Staphylococcus aureus* (S.A., Gram-positive) and *Pseudomonas aeruginosa* (P.A., Gram-negative) bacteria. Different loads of a few nanometer-sized Ag nanoparticles (<5 nm) on GO were prepared using various concentrations of a silver precursor (AgNO_3_) solution to optimize the Ag:GO ratio. Solutions containing 10–100 ppm of GO-Ag nanomaterial were compared for antibacterial efficiency. Our study offers an in-depth understanding of the role of smaller sized Ag loadings in the nanocomposite, further emphasizing its promising potential for higher antibacterial agents and possible biomedical applications. 

## 2. Results and Discussion

### 2.1. Structural and Morphological Properties of Graphene Oxide (GO) Nanosheets 

**Figure 1:**
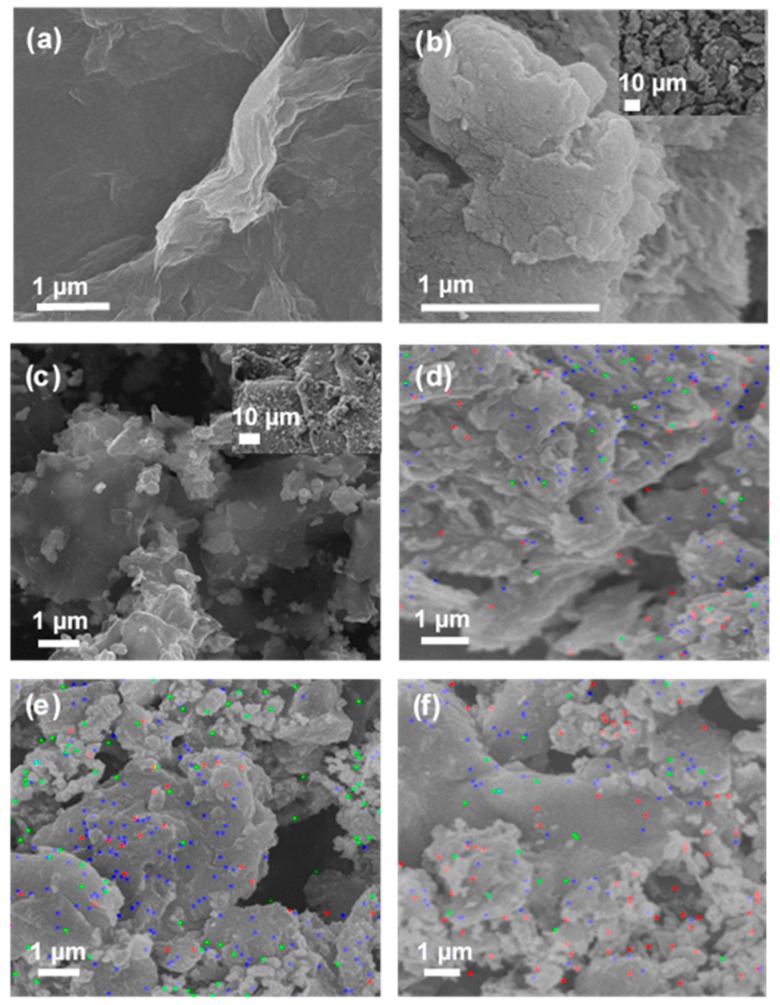
The field emission scanning electron microscope (FESEM) images of (**a**) pristine graphene oxide (GO); (**b**) thiol grafted graphene oxide (GO–SH); and (**c**) GO-Ag 0.2 M composites; FESEM mapping of (**d**) GO-Ag 0.1 M; (**e**) GO-Ag 0.2 M; and (**f**) GO-Ag 0.25 M composites. The red, blue, and green colors represent the elemental distributions of Ag, C, and O.

**Figure 2:**
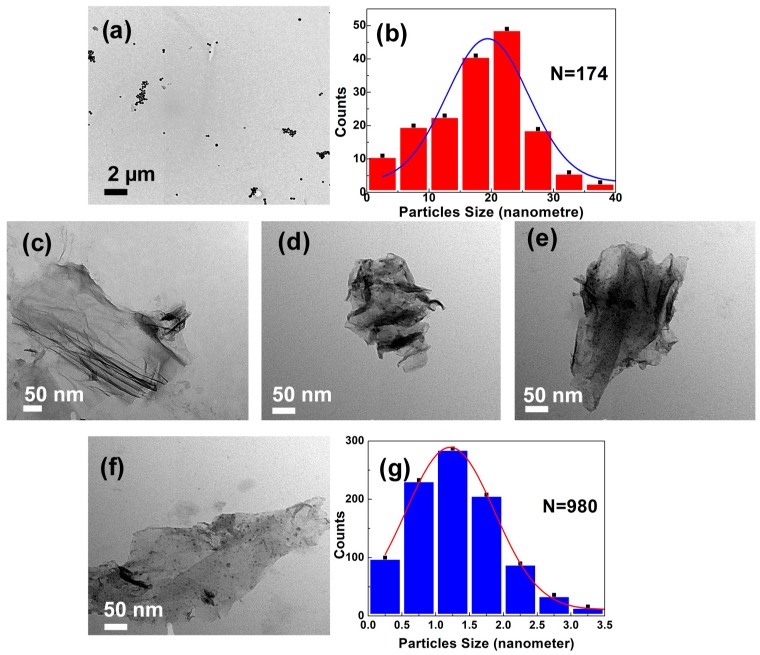
(**a**) Transmission electron microscope (TEM) images of pristine silver nanoparticles (Ag NPs) and their (**b**) particle size distributions without being grafted on GO; (**c**) pristine GO; (**d**) GO-Ag 0.1 M; (**e**) GO-Ag 0.25 M; (**f**) GO-Ag 0.2 M composites; and (**g**) the Ag particle size distributions of GO-Ag 0.2 M composites.

**Figure 3:**
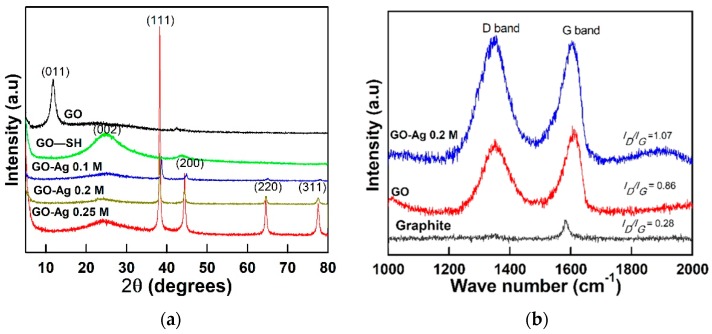
(**a**) The XRD graph of GO, GO–SH, GO-Ag 0.1 M, GO-Ag 0.2 M; and GO-Ag 0.25 M composites and (**b**) Raman analysis of graphite, GO, and GO-Ag 0.2 M composites.

The pristine GO was observed as wrinkled and wavy when dried in a vacuum oven as shown in Figure 1a. The corresponding transmission electron microscope image indicates that the GO had a flaky, smooth, and paper-like structure (Figure 2c). The GO average size was measured using the dynamic light scattering (DLS, Zetasizer, 2000 HAS, Malvern, Worcestershire, UK) technique. The average hydrodynamic diameter (AHD) of GO was recorded at 962.83 ± 141 nm (*n* = 3). The typical sharp X-ray diffraction peak (001) at 2*θ* of 11.7° confirms the formation of GO (Figure 3a) [8,24]. The calculated d-spacing of the GO is 0.76 nm. 

**Figure 4:**
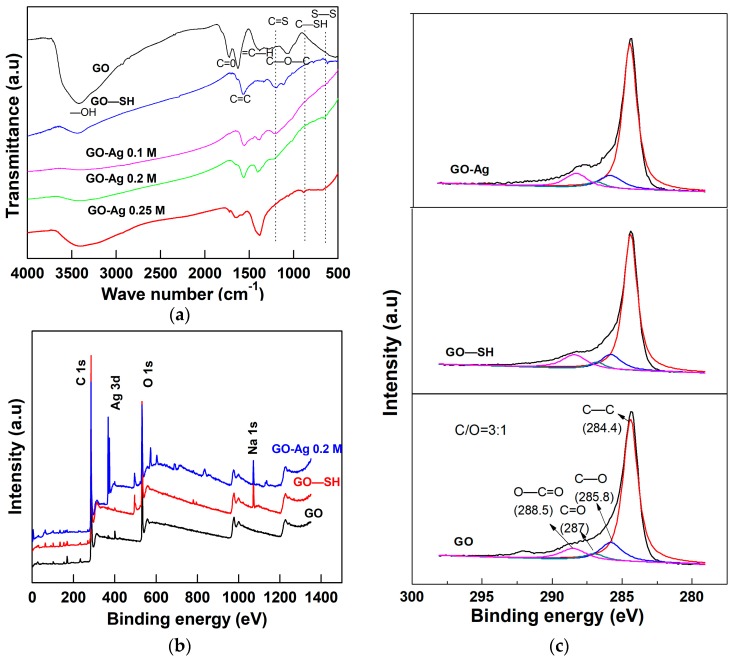
FTIR analysis of (**a**) GO, GO–SH, GO-Ag 0.1 M, GO-Ag 0.2 M, and GO-Ag 0.25 M composites; (**b**) XPS full scans of GO, GO–SH, and GO-Ag 0.2 M composites; and (**c**) C 1s deconvolution spectra of GO, GO–SH, and GO-Ag 0.2 M composites.

To analyze the carbon bond structure, Raman spectroscopy (Labram Hr800, Horiba, Ltd., Kyoto, Japan) was employed to examine the differences between the commercial graphite and synthesized GO samples. As shown in Figure 3b for the GO sample, the G band (1348 cm^−1^) is significantly higher than the D band (1583 cm^−1^) compared to graphite. When GO was oxidized from graphite, the *I*_D_/*I*_G_ ratio greatly increase from 0.28 to 0.86. The sp^2^ carbon bonding was broken by the oxidation process and transformed into sp^3^ bonding. Moreover, a Fourier-transform infrared spectroscopy (FTIR) analysis of the GO spectra (Figure 4a) indicated peaks at 3404, 1718, 1625, and 1055 cm^−1^ corresponding to the C–OH (hydroxyl), C=O, C=C (possibly due to the skeletal vibration of oxidized graphite domains), and the C–O stretching vibrations, respectively. The presence of those oxygen-containing groups (carboxyl, hydroxyl, and epoxy groups) confirmed the successful synthesis of the GO [24]. To further investigate the chemical structure, X-ray photoelectron spectroscopy (XPS) was utilized to study the bonding groups. Figure 4b,c revealed the overall and the C 1s deconvolution XPS spectra of the main GO’s bonding groups. The unique peaks attributed at 284.4, 285.8, 287, and 288.5 eV correspond to C–C, C–O, C=O, and O–C=O groups, respectively [25]. A full scan spectra of GO showed the C/O ratio around 3:1.

**Figure 5:**
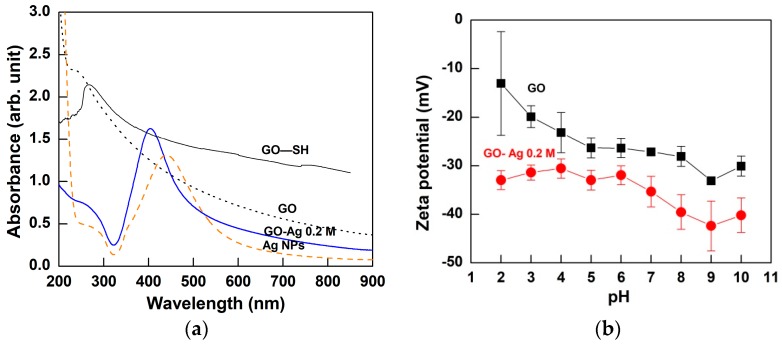
(**a**) UV-visible spectra of pristine GO, pristine Ag nanoparticles, GO–SH, and GO-Ag 0.2 M composite; (**b**) zeta potential profiles of pristine GO and GO-Ag 0.2 M composite.

UV-visible analysis of pristine GO was shown in Figure 5a. Typical peaks at around 230 and 310 nm correspond to the π-π* electronic transition of the C=C aromatic bonds and *n*-π* electronic transition of the C=O bonds [26]. The surface charge of GO ranged from 28.2 to 30.3 mV, indicating the moderate stability of the GO nanosheets as shown in Figure 5b [27]. In addition, there was a relative linear negative response of zeta potential of the GO as the pH value increasing from 2 to 10. This phenomenon is reasonable due to the effect of the carboxylic and hydroxyl groups ionizing causing an increase in the pH value. 

**Figure 6:**
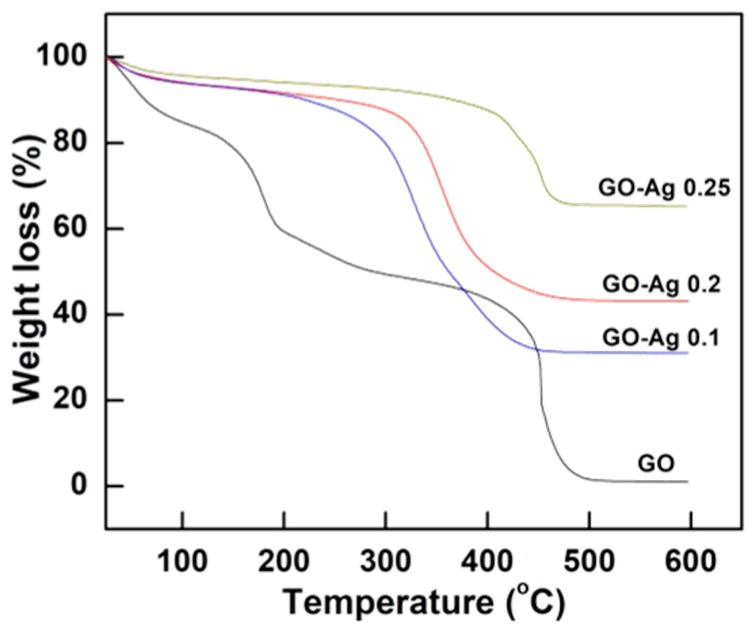
TGA weight loss of GO, GO-Ag 0.1 M, GO-Ag 0.2 M, and GO-Ag 0.25 M composites.

The pristine GO nanosheets were degraded in the air’s atmosphere as indicated in the thermal gravimetric analysis (TGA) as shown in Figure 6. The GO exhibited three stages of weight loss. The first peak dropped from 25 to 100 °C due to the removal of water from the remaining moisture. Other noticeable region weight losses were from 150 to 250 °C and from 400 to 500 °C. The former peak around 180 °C is ascribed to the removal of the oxygen functional groups from the GO’s surface while the other sharp peak around 450 °C is related to the burning of the carbon constituting graphene sheets [28]. The GO was completely degraded in the air flow at a temperature of 490 °C. 

### 2.2. Structural and Morphological Properties of GO–SH Particles

When NaSH reacted with GO, the nanosheets tended to be broken into many disoriented fragments during sonication and the stirring process. The GO–SH sample also has a sheet-like structure with agglomerations as shown in the field emission scanning electron microscope (FESEM) images (Figure 1b). The X-ray diffraction (XRD) spectrum of GO–SH sample shows the broadened peak at 2*θ* = 25° in Figure 3a which indicates the contribution of –SH group [29]. This was clearly confirmed by FTIR characteristic peaks at 1200, 620, and 838 cm^−1^ corresponding to the C=S stretch, S–S weak peak, and the secondary bond of thiol group C–SH bending, respectively (Figure 4a) [30]. The UV-visible spectra showed the obvious peak of absorbance in the GO–SH samples at 267 nm, which indicated the excited transition by capped-thiol groups at the terminated aromatic graphene oxide [31] (Figure 5a). Besides this, the XPS peak at 1071 eV confirms the appearance of sodium ions during the thiolation process (Figure 4b) [32]. The XPS analysis revealed that more O–C=O groups were formed in the GO–SH sample than the GO (13.2% versus 10.1%, Table 1). It was reported that the C 1s carbonate component (O–C=O) overlaps with adventitious carbon, increasing the ionization potential while having strong vibrations [33]. 

### 2.3. Structural and Morphological Properties of GO-Ag Composites

FESEM images and elemental mappings of GO-Ag samples with different AgNO_3_ concentrations are shown in Figure 1c–f. The Ag NPs were distributed on the GO nanosheets as primary nanoparticles. Moreover, the fine Ag NPs were attached on the GO sheets, indicating that there was strong bonding between GO–SH and the Ag NPs. The TEM images in Figure 2d–f indicated that the Ag NPs appeared as small dots on the GO nanosheets. The size distribution histogram of Ag NPs anchored on GO sheets was fitted with Gaussian curves to obtain the particle-size distribution (1.24 nm), as presented in Figure 2g. The narrow and uniform Ag NPs size on GO sheets is relatively consistent with the previous study with average size Ag NPs on GO sheets of approximately 2 nm [34]. The GO-Ag NPs showed a sharper UV-visible peak at 401 nm than pure Ag NPs at 443 nm (Figure 5a), indicating the successful Ag NP formation on the GO sample. This is in agreement with Lukman et al. [35] who reported 390–470 nm for Ag NPs, depending on the particle shape and size. The broad peak of 267 nm present in the GO–SH sample diminished in the GO-Ag nanocomposite, indicating that this thiol group was consumed during the Ag NPs formation. Without using NaSH and GO nanosheets, the synthesized particle size of the Ag NPs was much larger (19 nm) (Figure 2a,b). This confirms that the NaSH was efficient to reduce the Ag particle size. After attachment with Ag NPs, however, the mean size for the GO sheets dramatically reduced to 324 ± 20 nm (*n* = 3), which was significantly smaller than pristine GO (962 ± 141 nm). This may be due to breaking of the GO sheets into fragments via sonication, thus reducing the size [36].

The energy-dispersive X-ray (EDX) with FESEM mapping indicated that the Ag weight percentage increased for GO-Ag from 0.1 to 0.25 M. The Ag weight percent loaded on the GO sheets (as shown in Table 2) was 32.4, 44.3, and 62% using 0.1, 0.2, and 0.25 M of AgNO_3_, respectively. The GO-Ag composite shifted the main XRD peaks to 38.1°, 44.3°, 64.5°, and 77.5° in Figure 3a, which are assigned to the (111), (200), (220), and (311) crystal lattice planes of face-centered cubic Ag NPs, respectively. However, the typical GO peak at 11.7° disappeared in the GO-Ag samples due to the Ag NPs attached to the interlayers and covering the signals of GO peaks [37]. Prominently, the sharp peak at 38.1° confirmed the pure crystalline Ag NPs [38]. The Ag peak intensity increased steadily for GO-Ag from 0.1 to 0.25 M, reflecting different Ag contents in each sample. Besides this, Figure 3b shows the Raman spectra absorbance. After bonding with Ag NPs, the peak intensities increased with the G and D band at 1604 and 1354 cm^−1^, respectively. The ratio intensity of *I*_D_/*I*_G_ increased for GO from 0.86 to 1.07, indicating that a new defect was created during the Ag NPs formation process. In the GO-Ag FTIR analysis indicated in Figure 4a, the –OH groups become broadened while the intensity related to the bonding slightly decreased due to the interactions between Ag^+^ ions and the oxygen-containing groups on the GO sheets [39]. Remarkably, the peak of the C=C of sp^3^ clearly appeared in the GO-Ag samples at 1564 cm^−1^ during the Ag NPs formation attachment to GO sheets. The FTIR results confirm that the GO-Ag NPs, via grafting with the –SH group, were successfully synthesized. 

The XPS full scan analysis (Figure 4b) exhibited that the sharp peak at 368 eV confirmed that the Ag element was attributed to the GO-Ag sample. The C–C curve relatively increased while the O–C=O bonding was also partially reduced (from 13.2% in GO–SH to 11.7%, Figure 4c and Table 1) during the silver attachment process [40]. However, the content of carboxyl functional groups was higher in the GO-Ag composite than in the GO sample.

The increased carboxyl acid groups in the GO-Ag are attributed to the lower zeta potential values than the GO sample (Figure 5b). GO-Ag exhibits a higher negative charge due to the ionization of the multiple surface functional groups (–SH), and the products were dipped in alkaline solution during the synthesis process. This result also indicates that the chemical bonding with the thiol group modified the inherent surface zeta potential of the GO nanosheets, implying higher dispersion and stability in GO-Ag. Moreover, the zeta potential value of GO-Ag has less variation and more negative charge than the GO sample. This result indicated that GO-Ag is more stable than GO. 

TGA (Figure 6) was applied to analyze the weight loss at room temperature up to 600 °C in order to estimate the leftover Ag amount on the GO sheets [41]. The Ag content was 31% in GO-Ag 0.1 M while the percentages were 43% and 65% which correspond to the samples of GO-Ag 0.2 M and GO-Ag 0.25 M composites, respectively (Figure 6). 

Based on the above results, a mechanism was proposed to illustrate the chemical reaction process. Graphene oxide composed with the basal planes and the edges containing oxygen groups including epoxy, hydroxyl, and carboxyl groups [42]. Therefore, it is easy to disperse in water due to its high hydrophilicity, leading to its break-up into the fragments when it was sonicated for a long time. When NaSH was added, the site of –SH was anchored to the basal of the epoxy group due to orthogonal reactions of the –SH groups to selectively functionalize one site over another to form GO–SH. Thiolate GO may exhibit an in-specific binding capability towards the nanocrystal structure due to a high affinity towards Ag NPs [43]. On the other hand, the GO–SH mixture and an aqueous AgNO_3_ is embedded in a NaOH solution without any conventional reducing agents. The –SH groups acts as a binder and binds the Ag NPs to the GO through the phenolate anions of GO that are transformed into semiquinones [44]. With the presence of NaOH and the cross-linker GO–SH, the reduction reaction of Ag^+^ to form the Ag NPs was accelerated and oriented at the basal plane of the GO structure. Since we have fabricated a series of GO-Ag samples with various GO/Ag ratios, we will examine their antibacterial efficiency and search for the optimal GO/Ag ratio.

### 2.4. Antibacterial Results

#### 2.4.1. Antibacterial Activity at a Low Concentration of 10 ppm

**Figure 7:**
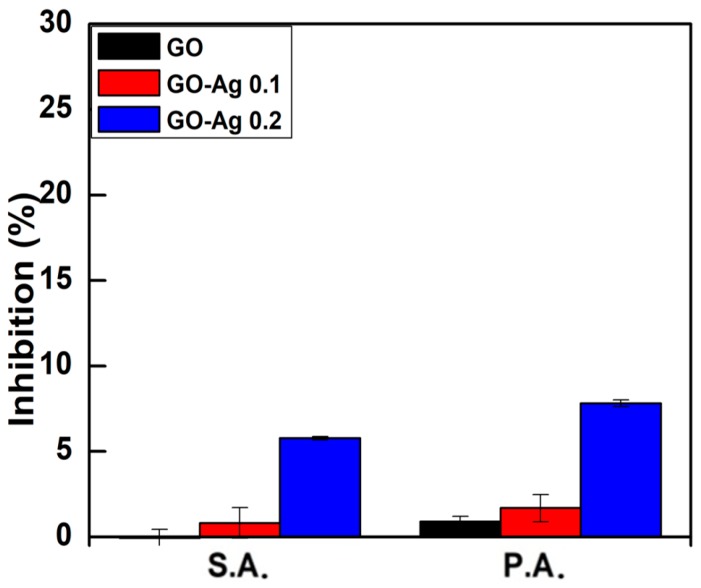
The inhibition percentages of the P.A. and S.A. bacteria treated with pristine GO, GO-Ag 0.1 M, and GO-Ag 0.2 M composites at a concentration of 10 ppm.

*Staphylococcus aureus* (S.A.) and *Pseudomonas aeruginosa* (P.A.) represent the positive and negative Gram bacteria used to measure the optical density (OD) with the 600 nm wavelength. At a low concentration of 10 ppm, Figure 7 showed that the number of bacteria was slightly increased proportionally to the AgNO_3_ concentration whereas almost no inhibition was observed in the GO sample. The inhibition of GO-Ag 0.1 M was 0.82% and 1.69% for S.A. and P.A., respectively. The GO-Ag 0.2 M had an inhibition efficiency of 5.79% and 7.82% for S.A. and P.A., respectively. Thus, the higher antibacterial activity of GO-Ag on P.A. rather than of S.A. may be due to the membrane structure difference. The gram-positive bacteria have a thick multilayered peptidoglycan of 20–30 nm while the Gram-negative one has a thinner membrane (8–12 nm) and is more vulnerable to antibiotics. This is the reason why S.A. is more resistant than P.A. [45]. 

#### 2.4.2. Antibacterial Activity at a Concentration of 20 ppm

**Figure 8:**
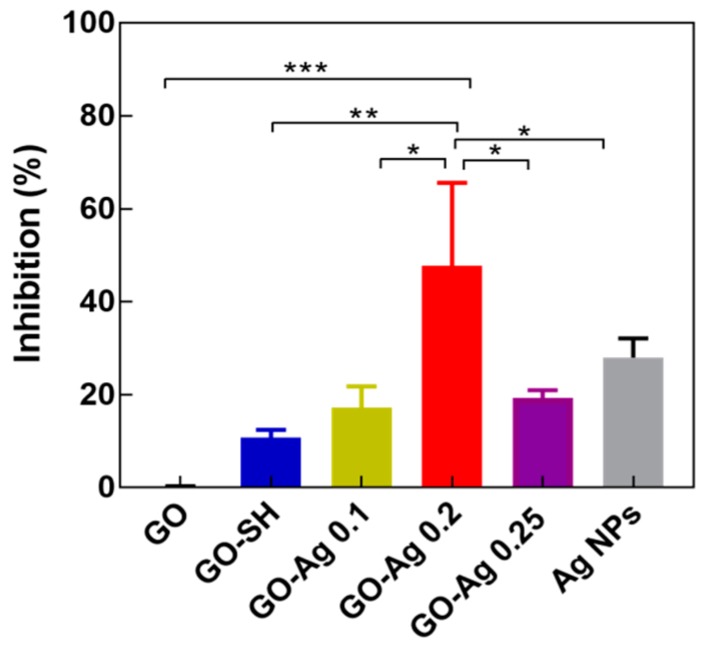
The inhibition percentage of S.A. bacteria treated with pristine GO and GO-Ag composite samples at a concentration of 20 ppm (*, ** and *** were represented for *p* < 0.05, *p* < 0.01, *p* < 0.001, respectively).

To investigate the contribution of the thiol (–SH) group to the GO-Ag composite formulation and the effect of Ag contents in GO-Ag NPs, a series of 20 ppm solutions were prepared for this test with S.A. bacteria. Figure 8 indicated that the GO had no inhibition (almost 0%) while GO–SH exhibited little antibacterial activity (10.8%), which was lower than the 0.1 M GO-Ag (16.7%) and 0.2 M GO-Ag (48.77%), respectively. Interestingly, we found that the inhibition of 0.25 M GO-Ag was reduced to 19.21%. Pure Ag NPs has an inhibition rating of 28.7%. This result showed that the Ag NPs may be the main factor which contributes to the antibacterial efficiency.

The higher Ag load in the 0.25 M sample did not benefit the antibacterial effect. The TEM images of Ag NPs in GO-Ag 0.25 M (Figure 2e) shows some Ag agglomerates, which may reduce the contact surface area of Ag NPs with bacteria. The GO–SH’s antibacterial function may be associated with the fact that the thiol group (–SH) reacted with GO to form a disulfide bond (S–S) which converts G–SH to glutathinone sulfide (G–S), causing oxidative stress to the cell [12,46]; especially when thiol was bonded with Ag NPs [47]. GO-Ag 0.2 M had the highest antibacterial capacity. The optimal Ag content was found to be approximately 42%, lower than the finding of Tang et al. (50% with 50 nm Ag NPs [48]). Since it has been reported that GO is the substrate that helps the mechanical immobilization meanwhile Ag NPs on the GO sheets will kill bacteria [48,49]. The relative amount between GO and Ag NPs needs to be balanced. The smaller sized Ag NPs in this work can reduce the effect of the Ag load for antibacterial treatment.

#### 2.4.3. Antibacterial Activity at a Concentration of 100 ppm

**Figure 9:**
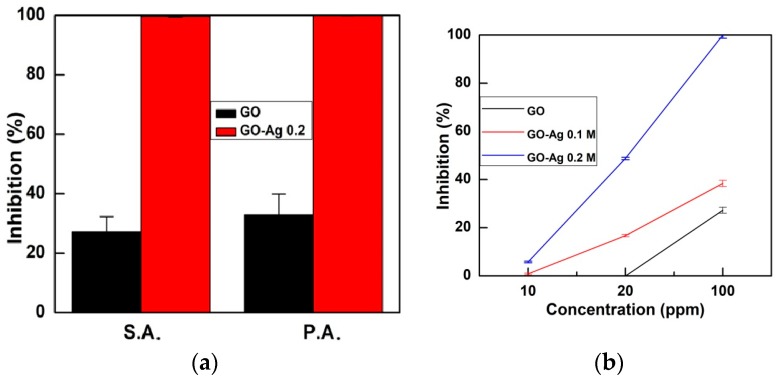
(**a**) The inhibition percentage of P.A. and S.A. bacteria treated with GO and GO-Ag 0.2 M samples at a concentration of 100 ppm; and (**b**) the antibacterial inhibition percentage of GO, GO-Ag 0.1, and GO-Ag 0.2 composites as a function of the load concentration against S.A. bacteria.

A high 100 ppm concentration of GO and GO-Ag 0.2 M composite was selected in further tests to compare their efficiency. The inhibition of S.A. (27.24%) was significantly lower than P.A. (32.86%) when using the GO sample. The GO-Ag resulted in an almost 100% inhibition (Figure 9a). It was reported that the GO performed the antibacterial effect at concentrations in the range of 10–500 ppm, depending on the oxidation level of the GO [8,12,24]. With the Ag NP modified GO, the inhibition concentration of the composites would be reduced. Moreover, Figure 9b shows that the bacterial inhibition level had a positive trend with the GO-Ag concentration of 10–100 ppm. This study demonstrates a promising effect for the antibacterial activity using appropriately designed GO nanocomposites at suitable concentrations. Further study is underway to determine the bacteria resistance and the associated gene expression of the bacteria in response to long-term exposure to the GO-Ag samples.

## 3. Materials and Methods

### 3.1. Materials

Graphite powder, sodium hydrosulfide (NaSH), and an ammonium hydroxide solution (NH_3_, 25%) were purchased from Sigma-Aldrich, St. Louis, MO, USA. Potassium permanganate (KMnO_4_) and trisodium citrate (Na_3_C_6_H_5_O_7_) were purchased from Nihon Shiyaku Industries Ltd., Osaka, Japan. The sulfuric acid (H_2_SO_4_) (95–98%) solution was purchased from Scharlab S.L., Barcelona, Spain. The silver nitrate (AgNO_3_) was purchased from Mallinckrodt Baker Inc., Paris, France and the hydrochloric acid (HCl) was purchased from Showa chemical co., Ltd., Honshu, Japan.

### 3.2. Bacterial Strains

S.A. (ATCC 25178) and P.A. (BCRC 12154) were obtained from the Bioresource Collection and Research Center in Hsinchu, Taiwan. The Difco^TM^ Nutrient broth and phosphate-buffered saline (PBS) were purchased from Sigma-Aldrich, St. Louis, MO, USA. 

### 3.3. Synthesis of GO

The GO was synthesized via a modified Hummer’s method [50] using graphite, H_2_SO_4_, and KMnO_4_ as the oxidizing agents. Around 2.4 g of graphite powder was dissolved in the 300 mL H_2_SO_4_ solution and stirred for 10 min. Subsequently, an amount of 2.4 g KMnO_4_ was added. Additional amounts of KMnO_4_ were added when the green color of MnO^3−^ diminished. A total of 5 equivalent weights of KMnO_4_ were sequentially added. After the MnO^3−^ was completely oxidized, 400 g of ice was added to the solution while keep in the ice bath to reduce the increasing temperature reaction. The solution was kept in the bath for a few days until the separation of the precipitation was clearly observed. The upper solution was removed, while the remaining precipitate was washed with deionized (DI) water in centrifugation (Hitachi, Tokyo, Japan) until the pH became neutral. The gel-like products were then dried at 60 °C in vacuum conditions overnight.

### 3.4. Synthesis of Ag NPs 

The Ag NPs were synthesized using trisodium citrate as a reducing agent by a modified Turkevich method [51]. In brief, 1 mM AgNO_3_ was dissolved in 60 mL DI water and continuously stirred at 200 rpm. Afterwards, the solution was vigorously boiling at 90 °C. Then, 6 mL of 10 mM trisodium citrate was added dropwise until the color of the solution turned into a bright yellow color. The final solution was allowed to cool at room temperature and then stored in a dark place for further usage. 

### 3.5. Synthesis of GO-Ag NPs

GO-Ag nanocomposites were synthesized by a slightly modified method from the reported literature [34]. An amount of 0.5 g GO powder was sonicated in 30 mL DI water for 20 min. Then, 8 g of NaSH was gradually added and the mixture was maintained at 55 °C with continuous stirring for 20 h. The product was filtered, washed with DI water and dried in a vacuum oven at 50 °C for 3 h. The collected GO–SH powder (0.1 g) was dispersed in 30 mL of DI water by sonication for 30 min. Subsequently, a series of aqueous solutions including 0.1, 0.2, and 0.25 M of 2 mL AgNO_3_ was added to thiolate the GO solution while stirring, respectively. Then, 0.1 M of 20 mL NaOH was added to the mixture and stirred for 20 h. The GO-Ag powder was obtained by centrifugation at 10,000 rpm several times and then dried in a vacuum oven at 60 °C for 24 h. The final dried powder was then filtered using dialysis tubing in order to remove the unreacted salt and loosely bound to Ag NPs. Different concentrations of AgNO_3_ in the GO nanosheets are referred so-called GO-Ag 0.1 M, GO-Ag 0.2 M, and GO-Ag 0.25 M composites, respectively.

### 3.6. Characterizations 

The microscopic images of the pristine GO, Ag, and GO-Ag composites were observed using a transmission electron microscopy (TEM, JEM 2000EXII, JEOL, Tokyo, Japan). In order to compare the distribution of particle size with total counts, the ImageJ software was employed to analyze the full width at half maximum (FWHM) based on the following formulation:

$$\bar{dn}=\frac{\sum_{i}Ni*di}{\sum_{i}Ni}$$

where *N*_i_ is the value of the frequency of counts or number of particles, *d*_i_ is the midpoint of classified size [52]. Additionally, the surface microstructure of the samples was determined using a field emission scanning electron microscope (FESEM) (JSM-7500F, Hitachi High-Technologies Corp., Tokyo, Japan) after the specimens were sputtered with gold. The elemental compositions were determined using an energy dispersive X-ray (EDX, Hitachi High-Technologies Corp., Tokyo, Japan) detector equipped with FESEM. The particle size distributions were measured using a dynamic laser light scattering analyzer (Zetasizer, 2000 HAS, Malvern, Worcestershire, UK) at room temperature in triplicates. An X-ray diffraction analyzer (XRD, model D5005D, Siemens AG, Munich, Germany) was used at 2*θ* ranging from 5° to 80° at a scan rate of 4°/min with Cu Kα radiation. The weight losses of samples were analyzed using a thermogravimetric analyzer (TGA, Model TA-TGA Q-500, TA Instrument, New Castle, DE, USA). Fourier transform infrared spectroscopy (FT-IR) (model Horiba FT-730, Minami-ku, Kyoto, Japan) was used to evaluate the functional groups of GO, GO–SH, and GO-Ag composites. The dried samples were calibrated and recorded in the range of 1000–2000 by a confocal micro-Raman spectroscope (Labram Hr800, Horiba, Ltd., Kyoto, Japan) at a wavelength of 785 nm under a power of 30 mW. A UV-visible spectrophotometer (V-650, Jasco, Hachioji, Tokyo, Japan) was used to measure light transmittance, and X-ray photoelectron spectroscopy (XPS K-Alpha, VG Microtech MT-500, Thermo Fisher Scientific Inc., Waltham, MA, USA) was used to examine the chemical composition of the samples. Sample suspensions in the cuvette were recorded for zeta potential in triplicates using a dynamic laser scattering analyzer (Zetasizer, 2000 HAS, Malvern, Worcestershire, UK) at room temperature after the pH value was adjusted using HCl or NH_3_.

### 3.7. Antibacterial Test 

The bacteria strain was kept overnight in a Difco^TM^ nutrient broth (NB) under aerobic conditions at 37 °C using a FIRSTEK S300R orbital shaker incubator (FIRSTEK S300R, Nankan, Taoyuan, Taiwan) for 12 h, centrifuged at 10,000 rpm for 5 min using Hermel (Z326K, New Taipei City, Taiwan). The supernatant was discarded and the precipitate was diluted with 12 mL PBS solution. About 450 µL of the above bacterial suspension was transferred into 48 well-plates. Then, 50 µL of each sample was added (control, GO, Ag NPs, and GO-Ag composites) and kept in an incubator for 1 h. Consequently, 10 µL of the sample mixture was taken out and transferred into 96 well-plates and again added 90 µL NB. The data was measured by a microplate reader (Biotek, Hong Kong, China) with an IP65LED light (Simon-Tech Inc., Nankan, Taoyuan, Taiwan) as a light source (with an optical density at a wavelength of 600 nm) during a 4 h period. The inhibition efficiency was calculated by using the following equation [53].

$$\mathrm{Inhibition}\;\left(\%\right)=\left(1-\frac{\Delta \;\mathrm{OD}\;\mathrm{sample}\;\mathrm{treatment}}{\Delta \;\mathrm{OD}\;\mathrm{control}}\right)\times 100$$

The distance between light and the culture plate was 20 cm and the light energy at that distance was 0.6069537 J/cm^2^ for a duration of 15 min. The experiment data were indicated by mean ± standard deviation (*n* = 3) and a one-way Analysis of Variance (ANOVA) using the GraphPad Prism 7 software to determine whether any significant differences remain between those groups or not. Therein, * *p*, ** *p*, and *** *p* were represented for *p* < 0.05, *p* < 0.01, *p* < 0.001, respectively.

## 4. Conclusions

The GO-Ag NPs, via grafting thiol groups, were successfully fabricated without using a stabilizer or a reducing agent. By adding AgNO_3_ solutions of various concentrations to GO suspensions, Ag contents of 31%, 43%, and 65% were obtained, with 1–2 nm sizes of Ag NPs anchored on the GO layers. During the synthesis process of GO-Ag, the graphene sheets were fragmented into smaller sizes. These GO-Ag samples have negative surface charges but the GO-Ag 0.2 M sample (43% Ag) demonstrates the highest antibacterial efficiency. At a 10 ppm load of GO-Ag suspension, only GO-Ag sample containing 43% of Ag yielded slight bacterial inhibition (5.79–7.82%). As the GO-Ag content was doubled to 20 ppm, the same GO-Ag composite exhibited ~49% inhibition. When the GO-Ag composite level was raised to 100 ppm, almost 100% of inhibition efficiencies were found on both *Staphylococcus aureus* (S.A.) *and Pseudomonas aeruginosa* (P.A.), which were significantly higher than using pristine GO (27% and 33% for S.A. and P.A.). The synthesized GO-Ag NPs show potential in both antibacterial and biomedical applications.

## Figures and Tables

**Table 1:** Bonding composition percentage of X-ray photoelectron spectroscopy (XPS) survey.

Samples	Carbon Bonding			
	C–C	C–O/C–S	C=O	O=C–O
GO	70.7	14.1	5.1	10.1
GO–SH	70.1	12.2	4.6	13.2
GO-Ag 0.2 M	72.5	11.2	4.6	11.7

**Table 2:** The energy dispersive X-ray spectroscopy (EDX) elemental composition (at %) for Ag NPs attached on the GO sheet layers.

Samples	C	O	Ag
GO-Ag 0.1 M	49.2	18.3	32.4
GO-Ag 0.2 M	38.5	17.1	44.3
GO-Ag 0.25 M	21.5	16.5	62.0
